# Examining the Impact of Patient-Reported Hope for Improvement and Patient Satisfaction with Clinician/Treatment on the Outcome of Major Depressive Disorder Treatment

**DOI:** 10.9734/INDJ/2016/26203

**Published:** 2016-05-09

**Authors:** Waguih William IsHak, Jennice Vilhauer, Richard Kwock, Fan Wu, Sherif Gohar, Katherine Collison, Shannon Nicole Thomas, Lancer Naghdechi, David Elashoff

**Affiliations:** 1 Department of Psychiatry and Behavioral Neurosciences, Cedars-Sinai Medical Center Los Angeles, California 90048, USA; 2 Department of Psychiatry and Biobehavioral Sciences, David Geffen School of Medicine at UCLA, Los Angeles, California, USA; 3 Department of Psychiatry, Emory University School of Medicine, Atlanta, Georgia, USA; 4 School of Public Health, University of California Los Angeles, Los Angeles, California, USA; 5 University of California Davis Law School, Davis, California, USA; 6 Western University of Health Sciences, College of Osteopathic Medicine, Pomona, California, USA

**Keywords:** Major depressive disorder, hope, patient satisfaction, remission

## Abstract

**Aims:**

This analysis aims at examining if patient-reported variables such as hope for improvement and patient satisfaction with clinician/treatment could influence the outcome major depressive disorder (MDD) treatment, namely depression remission, in the Sequenced Treatment Alternatives to Relieve Depression (STAR*D) trial.

**Study Design:**

Retrospective cohort study.

**Place and Duration of Study:**

The STAR*D study was conducted at 18 primary care and 23 psychiatric care settings in the United States from 2001–2007 and was funded by the National Institute of Mental health (NIMH). The analysis contained in this manuscript was conceptualized at the Cedars-Sinai Department of Psychiatry and Behavioral Neurosciences and performed at the UCLA School of Public Health.

**Methodology:**

Using data from STAR*D, the current study used logistic regression and survival analyses to examine the relationship between depressive symptoms remission and two sets of self-reported factors: Hope for improvement and, Patient satisfaction with treatment/clinician.

**Results:**

First, more than 90% of STAR*D patients reported having high hope for improvement (agree or strongly agree) and more than 66% endorsed high satisfaction with clinicians and more than 50% expressed high satisfaction with treatments (very or mostly satisfied). Second, hope for improvement was predictive of depression remission (p<0.05). Third, satisfaction with clinician/treatment, did not predict remission.

**Conclusion:**

This study shows the impact that patients’ subjective hope for improvement can have on predicting depression remission in contrast to satisfaction with clinician/treatment. Future studies should prospectively incorporate patients’ subjective attitudes regarding hope for improvement and satisfaction with clinicians and treatments as mediators and moderators of MDD treatment success.

## 1. INTRODUCTION

Psychiatric illnesses are increasingly recognized both by the medical and general population as chronic and disabling. According to the WHO, major depressive disorder (MDD) affects more than 350 million people worldwide [1]. MDD causes significant suffering due to symptoms of depressed mood, anhedonia, sleep and appetite problems, loss of energy and motivation, and suicidality. Moreover, impairments in quality of life and functioning compound suffering leading to loss of employment, inability to connect/care for loved ones, and higher cost to society [2–5]. MDD has grown to be of greater concern to society; it accounts for nearly a third of all non-communicable medical causes for disability. Despite extensive research, however, there remains a lack of clarity about the causes of depressive disorder and what antidepressants can and cannot achieve [6].

It is accordant, therefore, that one’s beliefs and attitude toward depression and its treatment might affect his/her outcome and tolerability of treatment [6]. Studies have discovered, for instance, that baseline satisfaction with medication is associated with improved clinical outcome [6]. This improved clinical outcome, in turn, mediates the effects of treatment on overall patient satisfaction [7].

Despite the detrimental effects of this cycle on patients suffering from depression, currently there is an unsatisfactory, considerably large gap of knowledge regarding the relationship between patient satisfaction (with clinician and treatment) and remission in major depressive disorder (defined as reporting no or minimal depressive symptoms, which corresponds to QIDS-SR=<5). Similarly, little research has been conducted regarding whether attitudes such as hope for improvement (defined as a patient’s ability to make important decisions and/or to enjoy activities of interest) can be predictive of remission. The goal of this paper is to investigate if remission (zero or minimal symptoms) can be predicted by patient satisfaction or subjective hope for improvement.

This study hypothesizes that depressed patients’ subjective hope to receive beneficial help from a doctor and/or patient satisfaction with clinician/treatment are predictive of remission from depression. It is important to note that the current study does not test the causal relationships of these variables and thus, the results and implications are that of association. These findings should inform the design of future research studies seeking to investigate causality.

## 2. METHODOLOGY

### 2.1 Study Population

The STAR*D study was conducted at 18 primary care and 23 psychiatric care settings in the United States from 2001–2007 and was funded by the National Institute of Mental health (NIMH). The study enrolled 4,041 treatment-seeking outpatients ranging from age 18 to 75 with a primary diagnosis of MDD [8]. To be eligible for the present analysis, participants were required to have complete data for each of the outcome measures detailed below, at both entry and exit for all four levels of the study. Patients who were in remission at entry of any level were excluded. The dataset analyzed in this study contained 2,280 participants in level one, 731 in level two, 188 in level three, and 54 in level four: all of whose data was taken from the original STAR*D dataset.

### 2.2 Treatments Administered

Treatment consisted of administering antidepressants sequentially, starting with citalopram for all patients in Level 1. In order to mimic clinical practice, the study used an equipoise stratified randomized design which allowed patients a choice between several switch or augmentation strategies, including cognitive behavioral therapy in Level 2. Medications were administered in fixed-flexible dosing and were modified based on response. Patients who did not achieve remission were moved to the next level. The four levels are summarized below:

**Table:** 

Level 1:	Citalopram monotherapy.
Level 2:	Either switching to sertraline, bupropion SR, venlafaxine XR, or cognitive behavioral therapy (CBT) OR Augmenting with bupropion SR, venlafaxine XR, or CBT.
Level 3:	Either switching to nortriptyline or mirtazapine OR Augmenting with lithium or triiodothyronine (T3).
Level 4:	Either switching to tranylcypromine OR Switching to venlafaxine XR + mirtazapine.

### 2.3 Outcome Measures and Analyzed Variables

The purpose of this study is to analyze pre-existing data gathered as part of the STAR*D Study to evaluate whether a patient’s subjective hope to receive help from a doctor and/or satisfaction with clinician or treatment is predictive of remission from depression. Remission was defined as no or minimal depressive symptoms, as evidenced by a score of 5 or less on the Quick Inventory of Depressive Symptomatology – Self Report (QIDS-SR) [9]. The QIDS-SR has scores ranging from 0 (not depressed) to 27 (most severely depressed). A score of 5 or less indicates remission, which is the goal of treatment. The QIDS-SR has high internal consistency (Cronbach’s alpha = 0.86) and is highly associated with the three versions of the clinician-rated Hamilton Rating Scale for Depression, as well as the Montgomery-Åsberg Depression Rating Scale and the Beck Depression Inventory [9].

In this study, hope for improvement comprised two patient-reported beliefs: 1) the belief that if one gets help from a doctor he/she will be better able to make important decisions (BETTER-DECISIONMAKING) and 2) the belief that if one gets help from a doctor, he/she will be better able to enjoy aspects of life (BETTER-ENJOYMENT). Similarly, patient satisfaction comprised two variables: 1) Satisfaction with own clinician (SATISF-CLINICIAN); and 2) satisfaction with treatment (SATISF-TREATMENT). The following four variables, collected during the course of the study, are the subject of this analysis:

1. BETTER-DECISIONMAKING (variable named MKEDC in STAR*D) refers to the following statement: “If I can get the help I need from a doctor, I believe that I will be much better able to make important decisions that affect my life and those of my family.”
2. BETTER-ENJOYMENT (variable named ENJOY in STAR*D) refers to the following statement: “If I can get the help I need from a doctor, I believe that I will be much better able to enjoy things that interest me.”
3. SATISF-CLINICIAN (variable named PSICL in STAR*D) refers to the following question: “How satisfied are you with the care currently provided by your Star*D study clinicians for the treatment of depression?”
4. SATISF-TREATMENT (variable named PSITX in STAR*D) refers to the following question: “How satisfied are you with the particular STAR*D treatment or treatments you are currently receiving for your depression?”

The statements included in the first two variables were rated on a Likert-type scale from 1 (agree) to 5 (strongly disagree), while the latter two questions were rated on a Likert-type scale from 1 (very dissatisfied) to 7 (very satisfied). The reliability of the above measures remains unknown as STAR*D was the first study to utilize these variables, and they have not been systematically examined. The first two variables measure patient-reported hope for improvement, and last two variables measure patient satisfaction.

### 2.4 Statistical Methods

The data was assessed for normality of distribution using the Shapiro–Wilk test. Summary values are expressed as means and standard deviations (SD) for continuous variables and frequencies (%) for categorical variables. For subjective hope of improvement variables, The Spearman correlation was preferred over the Pearson correlation because it better accounts for ordinal data. Spearman Correlation was used to measure the correlation between the BETTER-DECISIONMAKING responses to the Exit QIDS score. The same method was used to calculate the correlation between BETTER-ENJOYMENT and Exit QIDS score. BETTER-DECISIONMAKING and BETTER-ENJOYMENT scores were dichotomized into two categories: agree or disagree. Those who answered Strongly Agree or Agree were categorized in the Agree group. Those who answered neutrally (disagree or strongly disagree), were placed in the Disagree group. The next step was to count how many individuals in each of the two groups were at risk in each level. The percent of patients who entered remission and the percent that were censored were also analyzed. A patient was classified as “censored” if he/she never underwent remission; this means that the last observed QIDS score for that patient is not less than or equal to 5, and there is therefore no observed information to indicate that the patient underwent remission in the duration of the study. This can be due to either the study ending or the patient choosing to prematurely terminate his/her involvement in the study. “At Risk” individuals are categorized as the total numbers of individuals in the beginning of the level; this does not take into account patients yet to be classified as censored nor those who have entered remission in this level. A two-proportion z-test with pooled variances was used to test for differences in remission rates across all stages.

For patient satisfaction variables, the following procedures were utilized. Patient satisfaction with clinician scores (SATISF-CLINICIAN) were dichotomized into two groups: satisfied and dissatisfied. The responses “very dissatisfied”, “mostly dissatisfied”, “mildly dissatisfied”, “neutral”, and “minimally satisfied”, were placed into the “dissatisfied group.” The responses “mostly satisfied” and “very satisfied” composed the “satisfied group.” Similar steps were taken for the variable: patient satisfaction with treatment (SATISF-TREATMENT) scores. Since the sample size is large and n*p¬1¬ (1 −p2)>15, the data justifies a normally approximate z-test to measure the significance of the two groups. The current study’s statistician ran a logistic regression with the SATISF-CLINICIAN responses as predictors and the indicator QIDS remission as the response using PSICL1 as the reference group. This statistical analysis was conducted in order to determine if a patient’s responses regarding satisfaction with his/her clinician self-reported at the beginning of the level could help determine whether remission would be achieved at the end of each level. As a second approach, the SATISF-CLINICIAN scores were dichotomized by the options: “agree” and “disagree,” and a Fisher’s Exact test was run to determine whether patients who answered “agree” versus “disagree” had equally likely probability to achieve remission. The same methodology was performed with SATISF-TREATMENT responses to determine whether they may be predictors for remission. Analyses were performed using SAS software, version 10 (SAS Institute Inc, Cary, NC).

## 3. RESULTS

### 3.1 Study Population Demographics

At the point of entry, the analyzed sample used in this study, contained nearly two thirds women, one third college graduates, more than one half employed individuals, with the majority of patients being Caucasians (>80%).

### 3.2 Depressive Symptom Severity Scores

At Level 1 there were 2280 patients in the sample with a mean QIDS score of 9.53. As the levels progressed, the number of patients steadily declined, although there is an apparent general increase in the QIDS score as each level progressed. At levels 2 and 3 there were 730 and 188 patients in the sample with a mean QIDS score of 10.6 and 13.2, respectively. By Level 4 there were 54 patients in the sample with a mean QIDS score of 12. In other words, as each level progressed, there was a decreasing trend in the number of patients and an increasing trend in the QIDS score. This suggests that participants with lack of remission further into the study had worse QIDS scores, on average, to those earlier in the study.

### 3.3 Hypothesis 1: Hope for Better Important Decision Making and Better Enjoyment

Most of the patients appear to have a high subjective hope of receiving help from a doctor in the initial levels of treatment. That is, many patients at Level 1 “strongly agree” (975, 42.82%) that if they can receive help from a doctor, they will be much better able to make important decisions that affect their life and the lives of their family members (Table 1). Similarly, most patients “strongly agree” (1025, 45.02%) that if they can get the help they need from a doctor, they will be much better able to enjoy things that interest them (Table 2). By Level 4, the number of patients with these beliefs declined to approximately one-third of the original percentage (Tables 1, 2). Only 33.33% (18) patients responded with “strongly agree” in the BETTER-DECISIONMAKING questionnaire (Table 1), similar to the 31.48% (17) patients who did so in the BETTER-ENJOYMENT questionnaire (Table 2). The data also suggests that individuals who remain in the study longer seem to have a higher tendency to answer “agree” on their responses over any of the other answers (Tables 1, 2). In the BETTER-DECISIONMAKING questionnaire, the “agree” category had the most patient responses in each level (Level 1=48.48%, Level 2=50.14%, Level 3=48.94%, Level 4=62.96%).

Likewise, the “agree” category in the BETTER-ENJOYMENT questionnaire also had the most patients in each level (Level 1=47.65%, Level 2=47.67%, Level 3=46.28%, Level 4=59.26%).

Interestingly, patients who answered, “strongly agree” seemed to exit the study earlier as can be seen in Tables 1 and 2.

There seems to be no significant correlation between the two variables, seen in Level 1 (ρ = 0.01, P=0.719), Level 2 (ρ=0.00, P=0.978), Level 3, (ρ= 0.04, P=0.552), and Level 4 (ρ=0.04, p=0.757).

Similarly, there is no significant correlation between BETTER-ENJOYMENT responses and QIDS score; Level 1 (ρ = 0.03, P=0.107), Level 2 (ρ=0.00, P=0.992), Level 3, (ρ= 0.02, P=0.746), and Level 4 (ρ= −0.07, p=0.637).

In Level 1, there was a significant difference in the numbers of individuals who displayed remission between individuals who agreed (34.64%) and those who disagreed (25.13%) on the BETTER-DECISIONMAKING questionnaire (p<0.05). However, this difference was not significant as the treatment progressed to levels 2, 3, and 4 (p>0.05). There was also no significant difference in remission rates for individuals who agreed and disagreed with the BETTER-ENJOYMENT questionnaire at each level (p>0.05).

### 3.4 Hypothesis 2: Patient Satisfaction with Clinician/Treatment

In order to distinguish between patients’ “satisfied” and “not satisfied” reports, patient satisfaction scores were distinguished into two groups: agree and disagree. “Very dissatisfied”, “mostly dissatisfied”, “mildly dissatisfied”, “neutral”, and “minimally satisfied” were grouped into the “dissatisfied group.” Responses consisting of satisfied and very satisfied were placed in the “satisfied group.” The dichotomized SATISF-CLINICIAN and SATISF-TREATMENT values are presented in the Tables and Figures in their entirety (Tables 4, 5), respectively.

Initially, most individuals in the sample were satisfied with their clinician (79.45%, p<.001). As the levels progressed, however, satisfaction levels decreased (Level 2=72.87%, p<0.001, Level 3=66.67%, p<0.001). Despite the decrease, there were still significantly more individuals who answered a ‘satisfied” response than those who did not. In levels 2, 3, and 4, 20.55%, 27.13%, and 33.33% of individuals were dissatisfied, respectively. For the SATISF-TREATMENT questions, the opposite result is apparent. There does not seem to be a significant difference between the two responses given by individuals for any of the levels (p>0.05).

The data gained from a logistic regression model with SATISF-CLINICIAN responses as predictors and QIDS remission as the response indicates that the SATISF-CLINICIAN scores do not predict post-treatment QIDS scores at any level (p>0.05). This finding runs moderately in contrast to literature studying the importance of a patient’s relationship with his/her clinician as a factor in psychotherapy treatments, which generally report to a moderate correlation between outcome of the treatment [10] and [11]. Thus another approach we tried was to dichotomize the SATISF-CLINICIAN scores into “agree” and disagree” categories and run a Fisher’s exact test to see if patients that answer “disagree” and “agree” have equally likely probability to have remission. Again, results demonstrated SATISF-CLINICIAN responses do not predict and remission rates (P = 0.35, 0.82, 0.70 for Levels 2,3, and 4 respectively).

Regression analysis for SATISF-TREATMENT responses yielded similar results. SATISF-TREATMENT scores did not significantly predict exit QIDS score at any level (p>0.05). The Fisher’s exact test also did not show any significance in SATISF-CLINICIAN responses and remission rates (P = 0.87, 1.00, 0.45 for Levels 2,3, and 4 respectively).

**Fig. 1:**
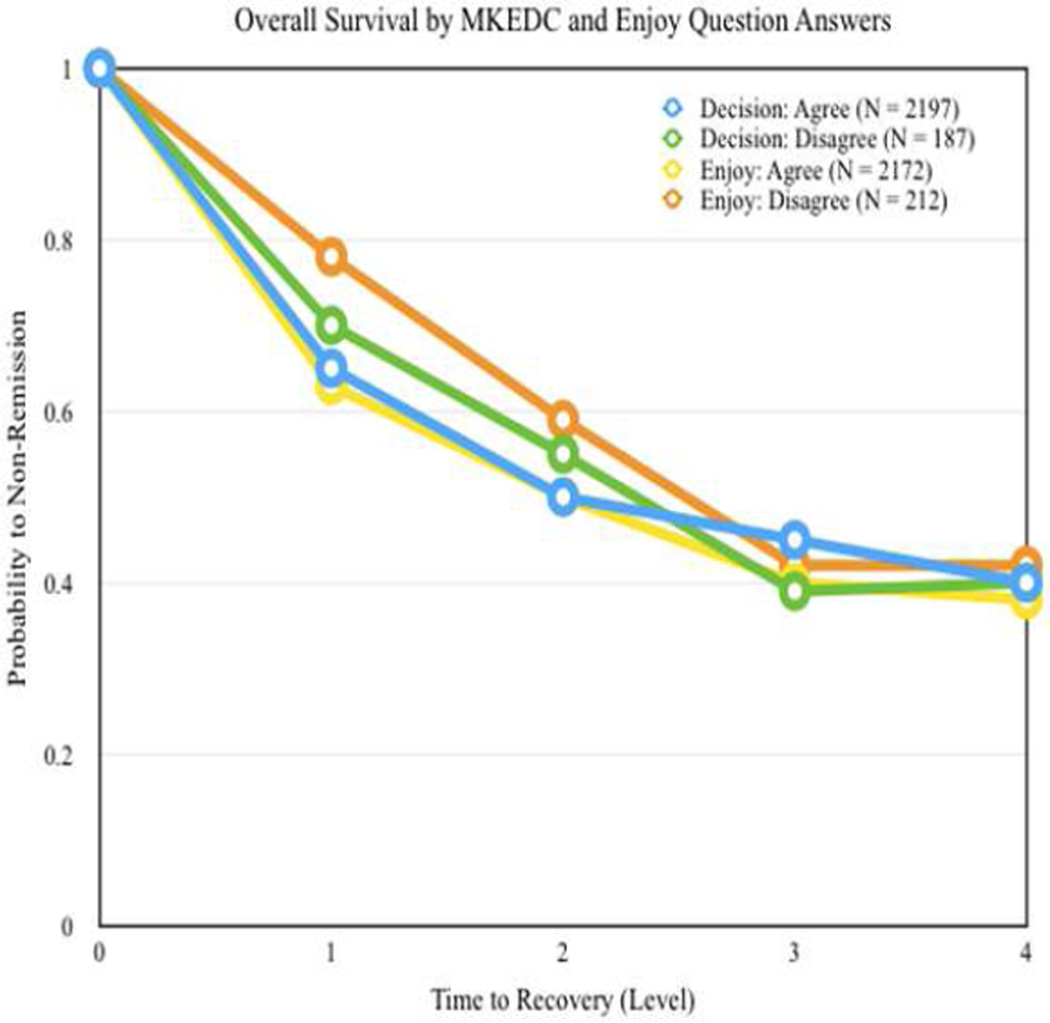
Kaplan meter survival curves for the BETTER-DECISIONMAKING and BETTER-ENJOYMENT groups

Overall the “agree” patients (N=2197) has a better remission rate than the disagree group. At level 3, this trend reverses, as the Kaplan Meier Survival Curve displays (Fig. 1). It is not, however, conclusive to rule that overall survival rate for disagree group is significantly better after level 3 since, as is indicated in Fig. 1, the sample size is very small for the disagree group at the later stages, which lowers statistical power for analysis.

It can be seen that the “agree” group has a better remission rate than the “disagree” group at each level, as the second Kaplan Curve portrays.

## 4. DISCUSSION AND CONCLUSION

The purpose of this study is to investigate whether remission could be predicted by patient-reported hope for improvement and from patient satisfaction with clinician/treatment. Results confirm that the hope for seeing a doctor to improve interest and enjoyment was predictive of remission. Patient satisfaction with clinician or treatment, in contrast, did not predict remission.

These findings contribute to an evolving picture of what variables are most important to clinical outcomes in the treatment of depression. Patients' expectations have long been considered a contributory factor to successful treatment [12,13,14,15], though they are also associated with the placebo effect in medication trials [12,13,14]. The finding that hope, defined as the result of positive expectations that the patient has about the treatment outcome, was predictive of remission is consistent with findings from the NIMH Treatment of Depression Collaborative Research Program [16]. Sotsky’s research revealed that higher patient expectation of improvement was significantly associated with a lower level of depression severity at termination of treatment, as well as a greater likelihood of complete response. It has been demonstrated that positive expectations can be altered in patients with MDD [17] by using structured cognitive techniques and that improving positive expectations can predict improvements in important outcome variables such as quality of life [18]. Considering the fact that patient perceptions of psychiatric treatment for depression are often poor, our finding highlights the importance of addressing the specific expectations a patient has for treatment and the continued need to develop methods to improve patient expectations for treatment [19,20,21].

Our findings that patient satisfaction with clinician or treatment did not predict remission is contrary to findings in some existing literature; previously conducted research indicates that baseline satisfaction with medicine significantly influences the outcome [6]. The effect of satisfaction with medication on a patient’s outcome is particularly noticeable in trials involving placebos. This finding may be due to the placebo effect, which accounts for 50% of the response to pain medication and 80% of the improvement with antidepressants [6]. We believe that further research into the placebo effect correlation would be an interesting and potentially illuminative addition to the current study; it is our recommendation that future studies expand on this effect to discover the impact of the placebo effect on patient satisfaction with medication.

Research has long since indicated alliance, defined as the relationship one has with their treatment provider, as an important variable in the treatment process. Such studies have gone so far as to posit alliance as a potential explanation for placebo effects in medication trials [22,23,13]. A recent study examining therapeutic alliance and treatment outcomes in patients with depression illuminates our finding that patient satisfaction with clinician did not predict remission. In this study, researchers measured alliance using the California Pharmacotherapy Alliance Scale [12]. Their results revealed that 3 of the 4 alliance subscales, including treatment provider understanding and involvement, were not related to change in depression scores, and only goal-oriented and working strategy consensus predicted symptom change. Another recent study found that a patient may be satisfied with their clinician, but this satisfaction did not lead to improved treatment adherence [24]. The only predictors of treatment adherence in the study were personality features and social support [24]. These findings suggest that a patient’s general satisfaction with a clinician may not be as significant to the treatment outcome as previously thought.

Our results may have differed from previously conducted studies due to the fact that we took a different approach than previous research. Rather than investigating whether clinical outcomes effect patient satisfaction, we examined whether patient satisfaction was predictive of clinical outcomes or, more specifically, remission. Our finding that satisfaction was not predictive of remission calls into question what role satisfaction plays in depression treatment. While satisfaction with treatment is, undoubtedly, an important goal of patient care, it is important that treatment studies continue to examine other important variables that may be significant contributors to clinical outcome.

Additionally, it is possible that the difference in findings is due to how the outcomes of treatment are operationalized. In our study, we studied remission as a post-treatment score: that is, no or minimal depressive symptoms as measured by the QIDS-SR score of 5 or less after treatment. For treatment outcome research, this perspective is indispensable. For clinical purposes however, the state of the patient at the end of treatment is equally important [25]. For instance, a patient may suffer form residual symptoms after treatment, but people’s perspective of outcome are often measured by the degree to which they reached their goals, rather than scores. The degree of score change indicates limited information about the state of patients at the end of treatment. A patient who has many residual symptoms might have improved during therapy as much as one who has few residual symptoms. However, these two individuals may feel very differently at the end of treatment [25]. Thus, individuals might find themselves quite satisfied with treatment if they reached their goal, yet this does not necessarily correlate with symptom remission. In addition, patients seem to base their satisfaction on absolute outcomes rather than relative ones. That is, patients tend to focus more on how they feel in the present, as opposed to how much their condition has changed [26]. Satisfaction, therefore, is not necessarily predictive of remission, especially if it is considered retrospectively. One’s treatment outcome expectations, in contrast, which is defined as one’s expectations about the consequences of undergoing therapy or undergoing specific therapy, can predict the presence of residual symptoms after treatment [27]. In conclusion, the results of this study reveal that a patient’s subjective hope for seeing a doctor to improve interest and enjoyment is predictive of remission. Satisfaction with clinician and treatment, on the other hand, was not.

As with all studies, this research is not without its limitations. We believe that by highlighting this study’s major limitations, we provide future researchers the opportunity to expand upon and enhance the accuracy of the current results. Firstly, there are overall limitations inherent in the STAR*D study and its dataset: most significantly, the lack of a control or placebo arm. Considering that this study utilizes a self-report method of obtaining patient data, it is important to highlight the second limitation; self-report threatens the accuracy of a study’s measurements. This could be accommodated for in future studies if researchers used methods for data collection that are potentially more precise, such as experimenter observation as opposed to patient self-report methods. Thirdly, an analysis of the patients who chose to terminate their involvement in the study early may prove beneficial. Future studies may benefit from a separate analysis of these participants whose attrition caused the sample size to suffer.

Lastly, this study does not test the causal relationships between depressed patients’ subjective hope to receive advantageous help from a clinician, their satisfaction with said clinician and treatment, and remission from depression. These findings must be interpreted as associations, as opposed to direct causes and effects. It is our hope, therefore, that the current study’s results and implications should, in turn, provide the foundation for future studies. These research studies may wish to investigate causality between these variables; perhaps by conducting and analyzing the results of a large-scale experiment with patients suffering from depression disorders.

## Figures and Tables

**Table 1:** Frequency tables for hope for better important decision making (BETTER-DECISIONMAKING) by levels

Level	Strongly agree	Agree	Neutral	Disagree	Strongly disagree	Total
1	975	1104	178	18	2	2277
	42.82%	48.48%	7.82%	0.79%	0.09%	100.00%
2	298	366	57	8	1	730
	40.82%	50.14%	7.81%	1.10%	0.14%	100.00%
3	84	92	12	0	0	188
	44.68%	48.94%	6.38%	0.00%	0.00%	100.00%
4	18	34	2	0	0	54
	33.33%	62.96%	3.70%	0.00%	0.00%	100.00%

**Table 2:** Frequency table for hope for better enjoyment (BETTER-ENJOYMENT) by levels

Level	Strongly agree	Agree	Neutral	Disagree	Strongly disagree	Total
1	1025	1085	155	12	0	2277
	45.02%	47.65%	6.81%	0.53%	0.00%	100.00%
2	315	348	64	3	0	730
	43.15%	47.67%	8.77%	0.41%	0.00%	100.00%
3	83	87	17	0	1	188
	44.15%	46.28%	9.04%	0.00%	0.53%	100.00%
4	17	32	5	0	0	54
	31.48%	59.26%	9.26%	0.00%	0.00%	100.00%

**Table 3:** BETTER-DECISIONMAKING and BETTER-ENJOYMENT’s correlation with QIDS score by level

	Level 1	Level 2	Level 3	Level 4
Spearman correlation: Decision	0.01	0.00	0.04	0.04
Spearman correlation: Enjoyment	0.03	0.00	0.02	−0.07
P-value: Decision	0.719	0.978	0.552	0.757
P-value: Enjoyment	0.107	0.992	0.746	0.637

**Table 4:** Frequency table for patient satisfaction with clinician (SATISF-CLINICIAN) by levels

Level	Very dissatisfied	Mostly dissatisfied	Mildly dissatisfied	Neutral	Minimally satisfied	Mostly satisfied	Very satisfied	Total
2	1	7	11	102	29	264	316	730
	0.14%	0.96%	1.51%	13.97%	3.97%	36.16%	43.29%	100.00%
3	3	4	4	30	10	59	78	188
	1.60%	2.13%	2.13%	15.96%	5.32%	31.38%	41.49%	100.00%
4	1	2	2	7	6	11	25	54
	1.85%	3.70%	3.70%	12.96%	11.11%	20.37%	46.30%	100.00%

**Table 5:** Frequency table for patient satisfaction with treatment (SATISF-TREATMENT) by levels

Level	Very dissatisfied	Mostly dissatisfied	Mildly dissatisfied	Neutral	Minimally satisfied	Mostly satisfied	Very satisfied	Total
2	24	53	48	180	60	217	148	730
	3.29%	7.26%	6.58%	24.66%	8.22%	29.73%	20.27%	100.00%
3	8	13	12	50	14	56	35	188
	4.26%	6.91%	6.38%	26.60%	7.45%	29.79%	18.62%	100.00%
4	4	1	0	21	3	12	13	54
	7.41%	1.85%	0.00%	38.89%	5.56%	22.22%	24.07%	100.00%
